# Physiologically Relevant Organotypic Tissue Slice Model for Evaluating Cell Responses to Ionizing Radiation

**DOI:** 10.3390/ijms27062850

**Published:** 2026-03-21

**Authors:** Victoria Shestakova, Ekaterina Smirnova, Elena Isaeva, Anna Smirnova, Dmitrii Atiakshin, Elena Yatsenko, Anna Yakimova, Sergey Koryakin, Denis Baranovskii, Vyacheslav Saburov, Yana Sulina, Lyudmila Komarova, Sergey Ivanov, Peter Shegay, Andrey Kaprin, Ilya Klabukov

**Affiliations:** 1 National Medical Research Radiological Center of the Ministry of Health of the Russian Federation, Koroleva st. 4, 249036 Obninsk, Russia; 2 Obninsk Institute for Nuclear Power Engineering, National Research Nuclear University MEPhI, Studgorodok 1, 249036 Obninsk, Russia; 3 Scientific and Educational Resource Center for Innovative Technologies of Immunophenotyping, Digital Spatial Profiling and Ultrastructural Analysis, Patrice Lumumba Peoples’ Friendship University of Russia (RUDN University), 117198 Moscow, Russia; 4 Institute of Systems Biology and Medicine, Russian University of Medicine, 111398 Moscow, Russia; 5 University Hospital Basel, Basel University, 4001 Basel, Switzerland; 6 Department of Obstetrics, Gynecology and Perinatal Medicine, Sechenov First Moscow State Medical University (Sechenov University), 119435 Moscow, Russia

**Keywords:** cell death, cell viability, ionizing radiation, organotypic tissue culture, organotypic tissue slices, radiobiology, radiation therapy, tissue engineering

## Abstract

Precision in radiotherapy requires the development of standardized, reproducible, and biologically relevant models to accurately assess the efficacy and safety of various radiobiological sources. This review presents a novel approach using precision-cut organotypic tissue slices (OTSs), or organotypic tissue cultures (OTCs), as a representative model with potential for unifying the assessment of radiobiological sources. Derived from specific organs, OTSs retain the complex architecture and multicellular environment of the tissue, providing a unique platform that bridges the gap between in vitro cell cultures and in vivo animal models. The typed OTSs can effectively mimic the in vivo physiological responses to ionizing radiation, providing insight into the mechanisms of radiation-induced damage and repair, and the potential for radiation-induced toxicity and side effects. The emerging practices for the use of OTSs in radiobiological studies include slice mechanical preparation, radiation exposure, and outcomes assessment. The prepared approach for OTS preparation promises to improve the reliability and comparability of radiobiological studies, facilitating the development of safer and more effective radiation therapies. OTSs have the potential to significantly advance our understanding and application of radiation medicine and research by providing a physiologically relevant assessment of radiobiological effects of novel ionizing radiation sources.

## 1. Introduction

Biological models for evaluation of radiobiological effects vary from simple biomolecular solutions to laboratory animals. Cell monolayers derived from cells obtained from tumor tissue biopsies and cellular spheroids (tumoroids) are used as routine models to assess the effects of ionizing radiation [1,2], primarily to evaluate cellular death and activity of repair mechanisms. Recent studies have increasingly focused on the influence of the tissue microenvironment of tumor cells, particularly the extracellular matrix. Unlike cell monolayers, ingrown tumoroids derived from cancer cells are capable of demonstrating a phenomenon known as “multicellular resistance” [3,4]. The mechanisms underlying multicellular resistance include suppression of apoptosis, a high proportion of quiescent cells, altered protein expression (particularly topoisomerases and repair enzymes), the presence of hypoxic and necrotic centers, and other mechanisms that may affect the efficacy of radiotherapy [5,6]. Since these mechanisms correspond to phenomena observed in vivo, the cellular 3D spheroids formed are considered relevant models for evaluating the effects of new ionizing radiation sources [4,5].

Cellular spheroids are formed from linear cells or primary human cells capable of not only forming cellular aggregates but also synthesizing the extracellular matrix (ECM) upon exposure to specific signaling molecules (e.g., TGF-alpha) [7]. Although the idea of using tissue spheroids to study the effects of ionizing radiation has been proposed before [7,8], it is only with the use of modern imaging analysis methods that it has been made possible to study these effects in detail.

The primary mechanism of action of ionizing radiation in the treatment of oncologic diseases is the stimulation of apoptosis in cancer cells [9]. Apoptotic cells exhibit morphological changes that typically result from the biochemical activation of caspases. However, recent evidence suggests that after exposure, cells can recover from complete apoptosis through a process known as anastasis, defined as the recovery of cells from the brink of apoptotic death [10]. This process allows cells to survive following caspase activation due to transient exposure to a lethal dose of an apoptotic stimulus [11]. Given that radiotherapy, chemotherapy and targeted therapy are often administered at high doses for short periods of time, anastasis may represent a survival mechanism that occurs in a subset of cancer cells, leading to chemoresistance and cancer relapse [12]. Cell recovery after caspase activation can also lead to genetic instability and increased mutational burden, as well as proliferation and migration, suggesting that anastasis may contribute to tumor progression and metastasis in addition to drug resistance [13].

Various models are used to evaluate cell death, such as monolayer cell cultures, which lack natural intercellular interactions that affect cell phenotype and regulation [14]. In addition, cell culture environment can influence cell phenotype and cellular responses to external environmental factors. Since cell phenotype and regulation are highly dependent on complex interactions with neighboring cells, the ECM, proteins, cell–cell and cell–ECM interactions differ in monolayer cultures, cellular spheroids, and between-cell layers in spheroidal structures, potentially affecting the results of metabolic activity assessments. Three-dimensional (3D) cell culture methods include cellular spheroids, hydrogel substrates, the hanging drop method, 3D bioprinting, macroporous scaffolds, etc. [15,16,17,18,19,20]. The absence of vascularization and normal diffusion limits spheroid size and stimulates ischemic or toxic cell death within the spheroid thickness [21].

The use of microfluidic devices, such as the organ-on-a-chip, allows for the simulation of the function of various tissues where the interaction between different cell types becomes critical [22,23]. However, these models have similar limitations to cell monolayers and cell spheroids. In particular, when assessing the effects of ionizing radiation, the role of the extracellular matrix and cellular microenvironment has a more significant effect on cells than paracrine interactions between tissues, so the use of microfluidic systems alone does not inherently improve the physiological relevance of the model [24,25]. Designing the experimental system to place the cell or tissue culture in a sealed volume enables convenient transport of the cultures without strict aseptic requirements and without the need to accommodate the geometry of a collimator.

Animal models remain a classic technique for investigating systemic responses in experimental radiobiology [26]. The use of animal models in radiobiology research facilitates the acquisition of physiologically relevant systemic outcomes and the imaging of changes at the tissue level, such as xenotransplantation in zebrafish or immunodeficient mice [27,28]. The key advantage of animal models is the ability to evaluate immune responses to ionizing irradiation, including immune cell subtype distribution and co-localization [29,30]. However, visualization of early radiation-induced complications typically requires the use of minimally invasive and microsurgical techniques for specimen collection, especially when using accelerated particle beams. Furthermore, in the absence of clear topographical landmarks, finding and identifying areas of interest during histological examination is a non-trivial challenge and can be easily distorted by individual anatomical or physiological variations in the animal.

The aim of this review is to justify the issue of standardization of biological objects to evaluate radiobiology effects of ionizing radiation sources.

## 2. Approaches for Handling Living Organotypic Tissue Slices

In the beginning of the 20th century, the famous scientists Alexis Carrel and Montrose Burrows performed basic investigations into the foundations of tissue culture [31,32]. Currently, physiologically relevant organotypic tissue cultures (OTCs), or organotypic tissue slices (OTSs), have not been fully developed, not only because of the difficulties in maintaining viability of a heterogeneous cell composition and maintaining physiologically relevant homeostasis, but also because of the inability to identify cellular events throughout the depth of the tissue using light and fluorescence microscopy techniques. The advent of confocal microscopy has made it possible to track events to depths of 500 µm, allowing the assessment of events in unfixed tissue sections.

Research on the mechanisms of cell death induced by ionizing radiation is currently focused on the problem of investigating the radiobiological effects of accelerated particles [33,34,35]. The use of fluorescent dyes enables the visualization of events related to alterations in cellular metabolism and cell death occurring as a result of exposure to ionizing radiation [36]. During fluorescent visualization of cellular events, morphologic assessment and visualization of cell viability in tissue sections can be performed using various methods, including phase contrast microscopy, live/dead assay, immunohistochemical analysis, transmission electron microscopy, scanning electron microscopy, multiphoton microscopy, and others. However, the live/dead assay is considered relatively crude because it relies solely on staining with Calcein AM and EthD-1 [37,38,39,40,41]. As an alternative, fluorescent dyes can be used, because their staining intensity varies with the mitochondrial membrane potential.

An alternative method for quantitative analysis of cellular activity in tissues is to determine the level of reactive oxygen species (ROS) in cancer cells. ROS are generated primarily in the mitochondria when electrons escape from the electron transport chain [42,43]. These electrons combine with molecular oxygen to form various reactive oxygen species. This method facilitates the visualization of ROS localization in adherent cells using confocal microscopy and also allows the determination of ROS intensity.

Cellular senescence and senescent drift are associated with specific cellular distribution and specific secretory phenotype [44,45], which are not related to the two-dimensional (2D) cell culture and physiologically irrelevant 3D cultures, which could be derived by cell seeding into the Matrigel scaffold [46]. Therefore, the use of physiologically relevant scaffolds or ECMs is critically important in order to investigate these parts of radiobiological effects.

Fluorescent dyes are traditionally used in cell culture, but the use of tissue culture requires more prolonged staining procedures to ensure the diffusion of dyes deep into the tissue. The question of whether fluorescent dyes, labeled antibodies, and other agents can penetrate deep into the tissue section without additional pumping of the medium through it under overpressure is discussed in the current literature [47,48,49,50,51,52]. In some cases, staining of tissue sections 200 μm thick may require a significant increase in exposure time, centrifugation, or staining under elevated pressure.

The influence of the ECM of both normal and tumor tissues on cell death and cellular regeneration (anastasis) remains poorly understood. Currently, the origin of cell anastasis is understood to be related to the inhibition of the cytochrome C release, disruption of cytosolic adaptogenic factors, and removal of damaged mitochondria to restore energy production, but the underlaying molecular mechanism remains unclear. Anastasis has been observed in several cultured human cancer cell lines, including cervical cancer, small cell lung cancer, neuroblastoma, skin cancer, testicular cancer, liver cancer, breast cancer, and prostate cancer [9,53,54,55,56], suggesting that this process may be a common phenomenon in cancer. Furthermore, the activation of genes involved in cell migration (MMP9, MMP10, and MMP13) and angiogenesis (ANGPTL4, ANGPT2, and VEGFA) during anastasis suggests a potential link between anastasis and cancer metastasis during recurrence [9]. On the other hand, the extracellular matrix exhibits protective functions when tumor tissues are irradiated. The development of a standardized model of surviving tissues will address many questions of resistance and will allow for the description of the influence of the extracellular matrix on cell anastomosis in tissues of different origins.

The creation of new ionizing radiation sources and radiopharmaceuticals and the evaluation of their efficacy require the use of physiologically relevant models. However, it appears that radiobiological differences may be less significant if appropriate models are selected and tested using clinically relevant exposures and protocols [57,58,59,60]. Despite significant clinical advances in the delivery of conformal radiation therapy and imaging, the risk of normal tissue complications continues to limit dose escalation and significantly impacts post-treatment quality of life [61,62].

The nature of OTSs allows for the investigation of radiobiological effects not only through visible light microscopy, fluorescence microscopy, immunohistochemistry (IHC), and histology, but also through genetic, transcriptomic [63,64], proteomic [65], and metabolomic studies [66]. Moreover, the mechanical integrity of OTSs also enables their heterotypic or orthotopic microsurgical implantation into laboratory animals as precision-cut solid tissue xenografts.

Therefore, the use of tissue models for the investigation of new methods in experimental nuclear medicine, radiotherapy and chemotherapy represents a current scientific challenge. The potential of cellular spheroids is limited by the heterogeneity of their composition and the absence of necessary macroenvironmental components (immune cells, microvessels, extracellular matrix properties), which does not always allow for the translation of such research results into the interpretation of effects obtained in animal models or clinical studies. The use of cultured tissue slices in conjunction with fluorescence visualization provides a physiologically relevant method for assessing the effects of ionizing radiation.

## 3. Current Approaches to 3D Tissue Models for Assessment of Ionizing Radiation Sources

Currently, there are several established techniques for radiobiological research, such as micrometer scale of tissue slices and unique cryo-focused ion beam milling, to produce frozen cell and tissue slices 100–250 nm thick, called ‘lamellae’ [67]. Classic microtomy and cryotomy techniques can produce tissue sections as thin as 1–1000 µm [68], but these methods do not provide live tissue sections suitable for assessing the effects of ionizing radiation. Living tissue samples must remain viable for approximately one month to assess apoptotic, necrotic, and proliferative changes in irradiated cells, and require the use of rotating tissue bioreactors, which are suitable for long-term tissue culture. However, this method imposes limitations on the linear dimensions of the samples—no more than 400 µm, limited by oxygen diffusion. At the same time, irradiating the samples requires a certain thickness sufficient to allow differential absorption of standard sources of ionizing radiation, such as gamma rays, electrons, protons, and neutrons.

The handling of 3D tissue cultures could be improved by using precision-cut ‘flannics’ as thin slices of biological tissue up to 200 µm thick, suitable for long-term culture and proposed for assessing the effects of ionizing radiation using laser confocal microscopy [69]. The organotypic flannic slices could be performed using the vibratome technique (vibrating blade microtomes) under standard conditions or with additional instruments and materials for mechanical support [70,71,72,73,74,75,76,77,78,79,80,81,82,83,84,85,86,87,88,89,90], listed in Table 1. 

**Figure 1:**
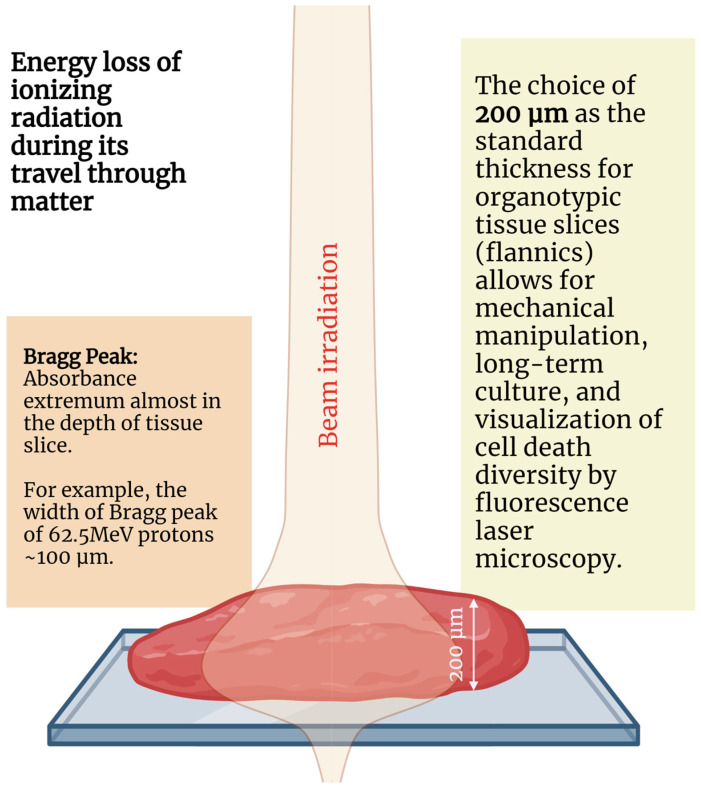
Organotypic tissue slices depth of 200 microns compiled for assessment of radiobiological effects of accelerated beams. Created with Bioender.com.

The spatial parameters of slices applicable for evaluating the Bragg peak effects of accelerated beams (Figure 1).

**Figure 2:**
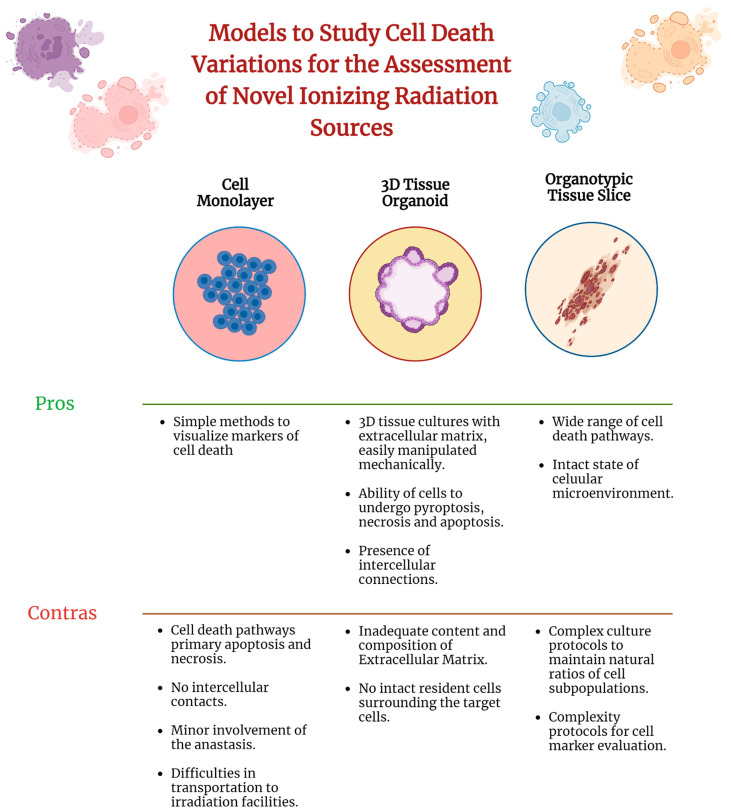
Comparisons of cell cultures, 3D spheroids, and organotypic tissue slices for the assessment of cell death pathways after ionizing irradiation exposure. Created with Biorender.com.

Spatial distribution of cells in tissues is regulated by the effects of extracellular microenvironments on cell regulation. The use of cellular monolayers is not physiologically relevant for the assessment of radiobiological effects associated with cell death diversity (Figure 2). Uprise of novel therapeutic accelerators based on carbon (C6+), helium (He2+), and iron (Fe26+) ions make important radiobiological effects in tissues with capabilities for live monitoring of these effects, enhancing precision in targeting tumors and minimizing damage to surrounding healthy tissues [91,92].

Radiobiological studies on 0.25–0.35 mm thick hippocampal slices shows both spontaneous and stimulation-induced neuronal activity after several hours of incubation [93]. Recently, the spectral characteristics of viable tissues were investigated based on 20 µm-thick tissue sections [94]. Kenerson et al. describe a comprehensive methodology for the manipulation of OTSs, which includes an initial step of immersing tissue samples in liquefied agarose gel [77,95]. However, the technique used to assess the viability of these sections using the MTS metabolic assay is not considered relevant. Currently, only the confocal laser microscopy toolbox could be used to evaluate cellular events in the depth of living tissues [96].

Therefore, the methods for assessing cell regulatory variants in 3D tissue cultures after irradiation should be performed using laser confocal fluorescence microscopy [97,98,99,100,101], which allows for restoring the volumetric distribution of cellular events in tissues (Table 2).

The application of these techniques requires that samples prepared using proposed protocols must maintain viability for at least one month to evaluate the apoptotic and necrotic responses and changes in proliferative activity of irradiated cells. The requirement necessitates the use of rotating tissue bioreactors suitable for long-term tissue culture. However, this method imposes an upper limit on the linear dimensions of the samples which should be no more than 400 µm, limited by the effectiveness for oxygen diffusion into the depth of living tissue (approximately 200 µm).

## 4. Tissue Cultures in Pharmacology and Radiobiology

The low success and effectiveness of clinical trials testing drugs in cell cultures, particularly 2D cell monolayers, has led to a reconsideration of the use of this technology and the development of new preclinical models that are capable of reproducing in vivo tissue physiology and microenvironmental factors. Although traditional 2D monolayer cell cultures are fundamental for high-throughput drug screening, the study of key molecular pathways, and the provision of vast insights into fundamental biological and disease processes, they have significant limitations [102,103]. Specifically, they lack critical three-dimensional (3D) interactions between cells and between cells and the ECM, which determine cell phenotype, drug penetration, metabolic gradients, and, ultimately, treatment response. This disconnect often results in a poor predictive value for in vivo efficacy and toxicity, a phenomenon famously illustrated by high attrition rates of compounds that fail in clinical trials despite promising 2D results [102].

To address these issues, pharmacologists have begun developing research in the field of cell culturing in three-dimensional (3D) systems. Cellular spheroids and tumoroids have become standard for studying drug penetration, tumor heterogeneity, and the microenvironment’s role in chemoresistance [20,104]. However, as it has been noted earlier, spheroids are intrinsically limited. Specifically, the absence of key stromal components (immune cells, fibroblasts, and vasculature) and the formation of spheroids from a single or limited number of cell types prevents a full reflection of the histoarchitecture and cellular diversity of the original tissues [105].

This makes the concept of precision-cut tissue slices, or ‘flannics’, key to pharmacology and radiobiology. However, tissue culturing differs from cell culturing due to valuable cellular heterogeneity, because the tissue culturing objective is not to expand cells, but rather to maintain existing cell populations and their physiological states. Given the significant differences in tissue origins, properties, and research objectives, we selected several tissue-culturing approaches to identify the key challenges (Table 3).

The comparative analysis of tissue culturing approaches, presented in Table 2, show the absence of a unified protocol for tissue culture cultivation. It is clear that key tissue culture parameters, such as the medium used, culture duration, incubation temperature, oxygen and carbon dioxide tension, are not universally applicable and they must be optimized depending on the tissue origin.

In particular, it is noted that, contrary to traditionally accepted culture conditions at 37 °C, incubation at lower temperatures (e.g., 32–36 °C) allows for the preservation of complex tissue architecture by reducing metabolic depletion [113,114,115,118]. We assume that a standard temperature of 37 °C can cause rapid metabolic exhaustion and faster degradation in thick tissue slices. This is clearly evident from the duration of cultivation and the decrease in viability in tissue sections, whereas slightly lowering the temperature preserves tissue architecture for weeks [113,114,115,118]. However, using low temperatures (<31 °C) leads to metabolic stalling, which prevents repair mechanisms and causes waste product accumulation [119]. A balance temperature of 32 °C will be safer than 36 °C for long-term survival.

Furthermore, hyperoxygenation (up to 70% O_2_) prevents the formation of a necrotic core in thick (>300 µm) slices [112] but leads to hyperoxia at the slice surface which can cause oxidative stress damage [120]. One advantage of using thick slices (~200 µm) is that it could eliminate the risk of hyperoxia at the tissue surface. Slices approximately 200 µm thick are sufficient to preserve the natural 3D microarchitecture, cell–ECM interactions, and stromal components, while still being thin enough to minimize severe diffusion limitations that would otherwise require extreme hyperoxygenation (>70% O_2_) in thicker slices [121].

In addition, cultivating tissue slices through an Air-Liquid Interface (ALI) allows for tissue preservation by simulating physiological oxygenation instead of full immersion, which can result in hypoxia in deeper layers [122]. A semi-permeable membrane is used to position the sites, which allows for the direct exposure of oxygen from the top as much as it allows for nutrients to be distributed from the bottom, resulting in an increase in cell viability and function [123]. The ALI approach stands out as particularly promising for epithelial tissues, such as skin, and also for flank tissues where the maintenance of a strict epithelial boundary is vital [124].

The comparative analysis shows that most tissue slice culture methods under standard conditions allow only for short-term pharmacological and toxicological studies [77,125,126], whereas assessing radiobiological effects often requires experiments lasting more than 10 days to predict delayed outcomes. Maintaining basic viability for a few days gives way to the need to preserve regenerative capacity, complex intercellular and cell–ECM interactions, and the production of signaling molecules and tissue-specific compounds for three weeks or more.

Thus, the development of 200 μm-thick tissue slice models, ‘flannics’, for radiobiological purposes, is a complex system requiring the determination of specific temperature regimes, O_2_ and CO_2_ concentrations, and ALI/perfusion support. Moreover, the composition of the culture medium is often determined by the tissue type being cultured and does not significantly affect tissue condition. Taking these factors into account will enable the use of OTSs not only as short-term ex vivo models for pharmacological and toxicological applications, but also as reliable, long-term platforms for radiobiological applications. This, in turn, will enable them to fulfill their intended role—to serve as a model system reflecting both the complex spatial structure of tissue and the temporal response to irradiation or the use of radiopharmaceuticals. In addition, this model will allow for the study of direct DNA damage, heterogeneity of cell death, as well as delayed responses of the tissue microenvironment and tissue-specific immunity.

Furthermore, the organotypic tissue slices’ unique features, such as preserved tissue architecture, multicellular composition, intact extracellular matrix, and tissue-resident immune populations, enable it to address clinically relevant questions about normal tissue complications, vascular injury, immune responses, fibrosis, and organ-level functional outcomes [68,116,127]. Specifically, human organotypic slice cultures have been used to study acute radiation injury and its subsequent mitigation by mesenchymal stem cell-derived extracellular vesicles [128], as well as to investigate drug–radiation interactions [63,129]. In other studies, organotypic slices have been identified as a promising platform that preserves receptor expression patterns and tissue architecture critical for assessing absorbed dose effects in targeted radionuclide therapy [130]. Thus, the use of organotypic cultures with advanced imaging techniques such as over-the-plane fluorescence microscopy (OTCxLSFM) enables the visualization of cell migration, invasion, and tissue remodeling at the single-cell level after irradiation [131,132,133]. However, the culture period in many protocols remains limited to a few days [134], which precludes the study of long-term effects of irradiation, such as fibrosis.

**Figure 3:**
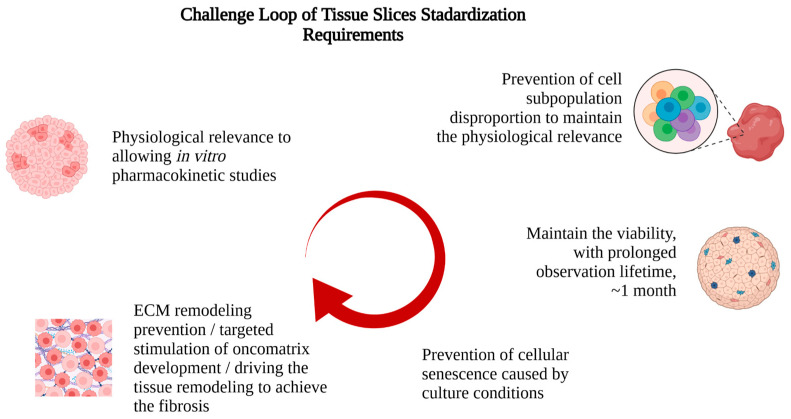
Keynote challenges for tissue culture and overcoming approaches. Created with Biorender.com.

The challenge loop of tissue slice properties using proposed protocols and approaches for their overcoming is presented in Figure 3.

Taking these factors into account will enable the use of OTSs not only as short-term ex vivo models for pharmacological and toxicological applications, but also as reliable, long-term platforms for radiobiological applications.

## 5. Future Research

The use of experimental models in radiobiology and the assessment of the efficacy of new sources of ionizing radiation, for example protons, heavy ions and combined radiation from radionuclides, has traditionally been limited to the properties of cell cultures, laboratory animals and, recently, cellular spheroids or tumoroids. The use of these models is not fully physiologically relevant, does not allow dynamic monitoring of physiological responses, or is characterized by heterogeneity and lack of a relevant microenvironment [135,136]. The precise assessment of individual responses to therapy in in vitro models is critical for predicting patient-specific outcomes [134], and researchers require advanced tissue-cutting techniques [137]

Currently investigated radiobiological effects go further than the evaluation of LD50 and double breaks into the evaluation of cellular senescence and senescence-related effects [138], investigations of the heterogenetic types of cellular death [139], and anastasis effects [140] which lead to cumulative effects into the immune microenvironment and ECM integrity. The curious task in radiobiology involves visualizing the radiobiological effects along the path of a radiation beam. The passage of accelerated particles through a substance generates a flux of secondary photons, electrons, and heavy particles, a phenomenon known as a track. It is not possible to evaluate cellular changes along this track using cell cultures or laboratory animals. All of these models are unable to quantitatively assess the production of secondary excitation during irradiation [141]. The OTS models will enable a comprehensive assessment of irradiation outcomes using fluorescent visualization of the different types of cell death (apoptosis, necrosis, etc.) to visualize the emergence along the entire track. Thus, the use of surviving tissue slices (tissue cultures) of normal and tumor tissues represents a physiologically relevant model that allows both in vivo and morphological studies of the effects of ionizing radiation, for example, effects of FLASH with carbon ions irradiation [142].

Tumors and normal tissue slices provide a powerful technique for evaluating many factors of tissue–drug or tissue–ionizing radiation interactions that cannot be studied in detail in either linear or primary cell cultures [143]. The use of tumoroids or cellular spheroids does not allow for the assessment of the effects of the natural extracellular matrix and is limited by the linear dimensions of such tumoroids. Therefore, the use of thin tissue slices that preserve the histoarchitecture of the studied tissue is of current interest.

The preparation of such slices requires specialized equipment, including tissue slicers capable of cutting the tissue into slices of a defined thickness. To preserve maximum viability for subsequent research, both the tissue-slicing parameters and the cultivation conditions should be carefully optimized. Furthermore, evaluating the effects on these slices necessitates the use of methods capable of visualizing cell viability throughout OTS tissue depth, which is critical for determining the biological effectiveness of novel ionizing radiation sources. Recent radiobiology studies have aimed to harmonize OTS derivation and handling protocols to ensure consistency in experimental conditions and radiobiological outcomes [125,128,130,131,133,144,145,146,147,148], including combinations of specific microscopy techniques and tissue-handling protocols [149]. Implantation into the Chick Chorioallantoic Membrane (CAM) could represent a potential approach for the long-term preservation of OTSs [150].

**Figure 4:**
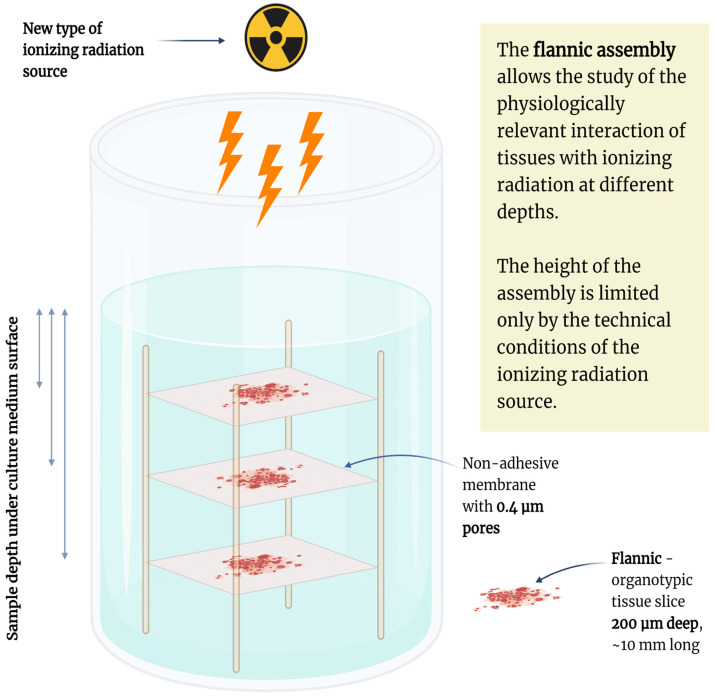
The use-case for the application of organotypic tissue slices for physiologically relevant assessment of the ionizing radiation sources. Created with Biorender.com.

We propose the use of a defined model for assessing the impact of ionizing radiation, which is known as the “flannic” model (“Fine-plane Native Tissue Cut,” or “flannic”), which was previously introduced for this purpose [69,151]. The properties of flannics culture are allowing for the assembly of multilayered structures for physiologically relevant assessments of radiobiological effects of ionizing radiation sources (Figure 4). The proposed construct is suitable for installation in T-CUP class flow culture systems [152] or advanced modular bioreactors.

The possibilities of obtaining cultivable and viable tissue slices could be explored for assessing the effects of different types of ionizing radiation, i.e., gamma rays, electrons, protons, neutrons, and heavy ions. The use of the presented flannic model assembly will allow for the visualization of the effects of ionizing radiation at various tissue depths. An important feature of such tissue assemblies is the potential for predicting and interpreting in vivo physiological effects, primarily monitoring double-strand breaks, vascular inflammation (vasculitis), heterogeneity of cell death mechanisms, inflammatory responses and cell–cell communications. The mechanical properties of the flannic tissue slices also allow not only for the investigation of cell features but also the derivation of decellularized scaffolds [153,154].

The 200 µm tissue slices have inherent limitations for assessing Bragg peak effects, as the distal fall-off of a pristine Bragg peak occurs over only tens of micrometers, a dimension smaller than the typical slice thickness. While clinical practice utilizes spread-out Bragg peaks (SOBP) rather than pristine peaks, and 200 µm slices cannot fully resolve the steep dose and LET gradients within the Bragg peak region, these models remain valuable for studying integrated biological responses across clinically relevant SOBP fields and for investigating out-of-field effects. Furthermore, the extracellular matrix and vasculature can be disrupted by mechanical cutting, which may lead to hypoxia and stress responses that affect baseline radiosensitivity and require controlling oxygenation.

## 6. Conclusions

Organotypic tissue slices of approximately 200 µm thickness represent a promising model for evaluating effects of all types of ionizing radiation including photons, electrons, neutrons, protons, and heavy ions. The primary goal of using these models is not only to preserve the native extracellular environment, but also to assess the mechanisms of cellular death caused by irradiation. The spatial scale of these slices makes it possible to evaluate the dose distribution of the Bragg peak in the depth of the tissue. These physiologically relevant tissue models could be used to compare results from different radiobiological studies.

## Figures and Tables

**Table 1:** Methods for obtaining 3D tissue cultures.

Cutting Methods	Peculiarities	Application	Ref.
Krumdieck tissue slicer	Classic cutting device; automatic slicer with a reciprocating blade. Requires pre-prepared cylindrical samples. Slice thickness ~100–500 µm.	Lung, liver, kidney	[70,71,72]
Vibrating Microtome	Oscillating (vibrating) blade in a buffer bath, frequency 85 Hz. Vibration reduces resistance, compression. A total of 50–1000 µm and deformation of soft tissue. Typical slices thickness: 50–1000 µm (standard ~200–400 µm).	Liver, solid tumors, brain, lung, heart, kidney, pancreatic tissue	[73,74,75,76,77,78,79,80]
McIlwain Tissue Chopper	Guillotine principle: a sharp blade is lowered onto stationary tissue fixed in an agarose block. This method is used to cut small or irregularly shaped specimens measuring ~100–1000 µm. Allows obtaining tissue cubes 300 × 300 × 300 µm.	Placenta, tumor, lung, liver, brain	[81,82,83]
Compresstome	A modernized version of the vibratome. The tissue is clamped between two plates during cutting, which minimizes vibration and damage. This method requires the tissue to be embedded in agarose. Typical slices thickness: 50–800 µm (standard ~200–400 µm). Producing sections approximately five times faster than Vibratome.	Brain, lung, tumor	[84,85,86]
Pulsed ultrafine water jet	Experimental method. Water is ejected by pressure from patch pipettes (10–12 µm tip diameters). Typical slice thickness: 450–500 µm.	Brain	[87]
3D printable tissue precision slicer and other do-it-yourself devices	A device created by assembling components using a 3D printer (Nylon-12). The system is suitable for long-term culture but requires tissue embedding in agarose gel. The device is operated by using manual mechanical cutting without vibration. Typical slice thickness: 200–500 µm.	Brain	[88]
Manual tissue slicing	Outdated approach, using a surgical scalpel, razor blades, or microsurgical instruments (tweezers, scissors) under a stereomicroscope. Typical slice thickness ~300–400 µm.	Brain	[89,90]

**Table 2:** Methods for assessment of cell regulation variants in 3D tissue cultures after irradiation by laser confocal fluorescence microscopy.

Staining Type	Description	Application for 3D Cultures	Ref.
MTT/MTS assays	The methods stain products of cellular respiratory metabolism.	The absorption line is very thin and non-standardized for 3D tissue slices.	[97]
Live/dead cell evaluation	The method is based on stained cells with damaged membrane and active intracellular enzymes.	The method could be compromised by auto-fluorescence of the extracellular matrix.	[98]
Double-strand breakage staining based on anti-γH2AX and other antibody-based techniques	Visualize double-strand breaks and other intracellular reactions.	The reactions could be studied during prolonged exposure and could be performed only by the nanobodies.	[99]
RNA-probes for evaluation of the cell expression	Fluorescent in situ hybridization (FISH) can help in understanding the spatial and temporal regulation of genes involved in neurogenesis, neuronal migration, and differentiation.	Additional physical or extended staining methods, cell membrane damage, or electroporation should be explored to perform the FISH technique for tissue slices.	[100]
Extracellular Matrix-targeted low molecular weight compounds	The methods could visualize the active groups in collagen macromolecules.	The method requires nanodots or low molecular weight fluorophores.	[101]

**Table 3:** Approaches for tissue culture across a wide range of conditions.

Tissue Origin	Physical Properties of Tissue	Media and Supplements	Physical Condition, Gas Media and Temperature	Features of Outcomes, Long-Terms Results	Ref.
Tumor Tissue (liver tumor)	Small (~250 μm) organotypic tumor slice cultures from patient-derived solid tumors. Preserves stroma, immune cells, and native ECM. Often highly vascularized or hemorrhagic.	Modified William’s E Media supplemented with hEGF (20 ng/mL), Nicotinamide (12 mM/mL), L-Ascorbic Acid 2-phosphate (175 μM/mL), Sodium Bicarbonate 0.225%, HEPES (20 mM/mL), D-(+)-Glucose (0.5%), Sodium Pyruvate (1 mM/mL), L-Glutamine (2 mM/mL). Penicillin Streptomycin (0.4%), ITS + Premix (1%)	37 °C, 5% CO_2_. Use a rocker (20 rocks/min) to alternately expose the sections to the medium and air.	Viability: limited to 5–10 days. Outcome: valuable for short-term assays of chemo-, immuno-, and cell-based therapies. Not suitable for long-term radiobiology requiring repeated assays.	[77]
Tumor Tissue (E.G7-OVA—mouse lymphoma; MC38—colon tumor C57BL/6 mouse)	Small (270 μm) tumor tissue slices.	Dulbecco’s Modified Eagle Medium (DMEM)/F12 Advanced supplemented with Fetal Bovine Serum (5%), 1 GlutaMAX (1%), Insulin-Transferrin-Selenium (1%) and HEPES (15 mM/mL). Penicillin/Streptomycin (0.5%),	37 °C, 5% CO_2_. Use a rocker (25 rocks/min)	Viability: limited to 7 days, optimal—48–72 h. Outcome: valuable for short-term testing immunotherapeutic agents, tumor-specific lymphocytes and CAR-T cells.	[106]
Brain Tissue (Hippocampus, Cortex)	Small (300 μm) tissue slices. Soft, layered, electrically active. High metabolic rate. Delicate synaptic architecture.	50% minimal essential medium, 25% horse serum, 25% Hanks’ balanced salt solution supplemented with 133 mM glucose, and antibiotics	37 °C, 5% CO_2_.	Enables long-term (weeks) electrophysiological recording and stimulation of live neural networks. Mimics functional connectivity for studying plasticity, epilepsy, and neurodegeneration in a preserved architectured context.	[93]
Brain Tissue	Organotypic brain slices, typically 100–400 µm thick.	50% MEM/HEPES, 25% heat-inactivated horse serum, 25% Hanks’ solution is supplemented with NaHCO3(2 mM), glucose (6.5 mg/mL), glutamine (2 mM). pH 7.2.	37 °C, 5% CO_2_.	Slices reorganize and thin but preserve original multilayered cytoarchitecture, specific neuronal connections (e.g., trisynaptic loop in hippocampus), and functional properties for several weeks to months in culture. Allows long-term studies of neurodegeneration, plasticity, and infection.	[107]
Liver Tissue (Precision-Cut Liver Slices)	Soft tissue slices, approximately 5 mg in weight, 250 µm thick. Maintains a sinusoidal network	William’s E Medium supplemented Human AB serum (5%), Glutamine (2 mM), Insulin (10 mg/L), Transferrin (5.5 mg/L), Sodium selenite (6.7 µg/L), EGF (1 nM), Glucagon (100 nM), Corticosterone (1 µM), and antibiotics.	37 °C, 5% CO_2_, 95% O_2_. Use an orbital rocker incubator at 70 RPM.	Viability: limited to 5–6 days, optimal 24–48 h. Outcome: suitable for short-term assays of toxicology and drug safety screening, liver fibrosis processes and regeneration mechanisms.	[108]
Retina–Retinal Pigment Epithelium	Small (250 μm), maintains the laminarity of all 10 layers	Dulbecco’s modified Eagle’s medium (DMEM), 15% porcine serum, 2.5% HEPES-buffer solution, 1% penicillin/streptomycin.	Perfusion culture: Use a two-compartment perfusion container. Gas-permeable silicone tubes, internal diameter 1 mm, perfusion speed: 1 mL/h, flow drive: peristaltic pump, 37 °C. Static culture: 37 °C, 95% air, 5% CO_2_, 100% humidity	Viability: limited to 10 days, optimal 24–72 h. Outcome: suitable for short-term assays of pharmaceutical agents or vitreous substitutes	[109]
Myocardium (Living Myocardial Slices)	Small (300 μm) soft tissue slice. Maintain 3D architecture with intact cellular interconnections and cell–cell junctions.	Medium-199 supplemented with penicillin–streptomycin, insulin-transferrin-selenite and 2-mercaptoethanol	37 °C, 5% CO_2_, 20% O_2_, 80% humidity. Use a rocker (60 rocks/min) and biomimetic cultivation chambers.	First protocol for reliable cultivation of human atrial slices for 1–2 weeks under near-physiological load. Allows direct modeling of atrial tachyarrhythmia, study of the impact of atrial arrhythmias, including atrial fibrillation, on structural and electrical remodeling, and drug testing.	[110]
Liver Tissue (Precision-Cut Liver Slices)	200–300 µm thick slices, cut with a mechanical slicer.	Williams’ medium E supplemented with n-glucose (25 mM), FBS (5%), insulin (0.1 µM), gentamicin (50 mL), saturated with carbogen at 37 °C.	37 °C, 40% O_2_, 5% CO_2_, Use a rocker Platform (~10 cycles a minute).	Viability: limited to 24 h, optimal 1.5 h. Outcome: maintains intact morphology, testosterone and lidocaine metabolism, and the ability to metabolize antipyrine at the same rate as after 1.5 h of incubation. The preferred system for long-term metabolism and toxicology studies on liver slices.	[111]
Liver Tissue	250 + 50 µm	Williams’ medium E supplemented with FCS (10%), BSA (0.2%), insulin (5 mg/mL), nicotinamide (10 mmol/L), hydrocortisone (1 mmol/L), penicillin (25 IU/mL), streptomycin (25 mg/mL).	37 °C, 70% O_2_ 25% N_2_ 5% CO_2_. Use a rocker platform (5 rocks/min).	Viability: 72 h. Outcome: high oxygen tension (70% O_2_), provides optimal conditions for preserving both morphological and biochemical integrity, without inducing significant oxidative stress.	[112]
Nervous Tissue	400 µm thick hippocampal slices	50% MEM/HEPES, 25% heat-inactivated horse serum, 25% Hanks’ solution supplemented with glucose (6.5 mg/mL). pH 7.2	36 °C, 5% CO_2_.	Viability: more than 6 weeks. Outcome: preservation of the layered structure of the hippocampus was observed. Pyramidal neurons exhibited typical morphology with developed dendritic spines and synaptic contacts similar to those seen in vivo. Analysis of excitatory and inhibitory synaptic potentials was retained. Active growth of new synaptic contacts and neuritic growth cones was observed during the first days of culture.	[113]
Skin Tissue (Split-Thickness Skin Grafts and Foreskin)	Explants (1.5 × 1.5 mm). Epidermal stratification preserved in culture.	Eagle’s minimum essential medium, 35% calf serum, Heparinized chicken plasma + 20% chick embryo extract (1:1 ratio).	32 °C, 95% air, 5% CO_2_	Viability: more than 3 weeks. Outcome: the peak of mitotic activity is delayed until day 3. The initial thickening of the epidermis is subtler, with less parakeratosis. An extended adaptation phase occurs, during which the epidermis is preserved, and 4–8 spinous layers are still visible on days 13–14. Epidermal viability is significantly prolonged, and active proliferation is maintained due to modulation of cell cycle kinetics.	[114]
Skin Tissue (Abdominal Wall of Cadavers or Breast Skin or Skin from Amputated Limbs)	Squares 3–5 mm squares from a 0.4 mm thick keratotome slice. Epidermis stripped.	Eagle’s Minimum Essential Medium supplemented with L-glutamine (1%), and calf serum (10%), penicillin G (200 U/mL) and streptomycin sulfate (100 eg/mL).	31–32 °C, 40% O_2_ or 20% O_2_ 5% CO_2_,	Viability: ~3 weeks. Outcomes: 1. Low culture temperatures slow epidermal proliferation, slow the rate of metabolic decline, prolong the adaptation phase in culture, and preserve tissue structure over a long period. 2. Elevated oxygen concentrations (40%) lead to increased proliferative and mitotic activity, especially in intact skin samples, where the stratum corneum limits diffusion. However, this leads to disorganization and the formation of cellular debris due to oxidative stress, leading to tissue integrity loss. 3. Oxygen concentrations below 20% lead to decreased viability and proliferation, potentially accelerating culture deterioration.	[115]
Brain Tissue (Hippocampus, Temporal Cortex)	300 μm thick	Organotypic slice culture medium (OSCM) with no serum consisted of additional energy substrates and metabolites.	37 °C, 95% air, 5% CO_2_	Viability: 3–4 weeks. Outcomes: suitable for long-term studies. Use of an OSCM may reduce astrocyte proliferation, the formation of exuberant synaptic connections, neurogenesis and network reorganization in culture, ambient levels of glutamate and toxic degradation products, thereby ensuring neuronal survival rather than proliferation.	[116]
Cheek Pouch Mucosa	Fragments ~1 mm^2^ Tissue maintained in organotypic relationship between epithelium and lamina propria.	Eagle’s Minimum Essential Medium supplemented with calf serum (10%), hydrocortisone (1 μg/mL), ascorbic acid (300 μg/mL), penicillin (100 I.U./mL), streptomycin (100 mg/mL) and amphotericin B (0.25 mg/mL).	37 °C, 10% CO_2_,	Viability: 23/24 cultures viable at 35, 42, and 49 days. Outcomes: the stratified squamous epithelium retained its normal maturation sequence, with a predominantly parakeratinized surface. Normal epithelial–connective tissue relationships were preserved, and the basement membrane remained clear and intact. There was no significant epithelial growth from the explant margins. This system is suitable for long-term studies of tissue metabolism, exposure to exogenous agents, and chemical carcinogenesis.	[117]
Adipose Tissue	Fragments 500 μm thick	DMEM supplemented with insulin-transferrin-selenium mixture (1%), Penicillin/Streptomycin (1%), Fetal bovine serum (10%).	Cultivation on a liquid-air-interface. 35 °C, 5% CO_2_	Viability: 14 days. Outcomes: 500 μm thick slices and low culture temperatures preserve adipocytes, macrophages, and stromal cells as well as maintain proliferation even without serum.	[118]

## Data Availability

The raw data supporting the conclusions of this article will be made available by the authors on request.

## Abbreviations

The following abbreviations are used in this manuscript:

CAM	Chick Chorioallantoic Membrane
ECM	Extracellular Matrix
LD50	Median Lethal Dose
OTC	Organotypic Tissue Culture
OTS	Organotypic Tissue Slices
ROS	Reactive Oxygen Species
